# Supplementary material for: Modelling bacterial twitching in fluid flows: a CFD-DEM approach
Source: Sci Rep. 2019 Oct 10;9:14540. doi: 10.1038/s41598-019-51101-3 (PMC6787227; doi:10.1038/s41598-019-51101-3)
Supplement: Supplementary file 1 — Supporting Information [file 41598_2019_51101_MOESM1_ESM.pdf]

# Supporting information

## Modelling bacterial twitching in fluid flows: a CFD-DEM approach

Pahala Gedara Jayathilake[1], Bowen Li[2], Paolo Zuliani[2], Tom Curtis[1], Jinju Chen[1]

[1]School of Engineering, Newcastle University, United Kingdom, NE17RU

[2]School of Computing, Newcastle University, United Kingdom, NE17RU

**Table S1.** Parameters used for the simulations.

| **Parameter** | **Value** | **Ref.** |
|---|---|---|
| Flow chamber | 50×20×20 $\mu m^3$ | - |
| Fluid shear rate | 0-200 $s^{-1}$ | (1) |
| Fluid Kinetic viscosity | $1\times10^{-6}$ $m^2/s$ (*water*) | - |
| Bacteria size | 5×1 μm, length × diameter | (2) |
| Bacterial and surface stiffness | $3.2\times10^{-5}$ N/m | - |
| Bacterial mass density | 1100 $kg/m^3$ (*chosen slightly bigger than that of water*) | - |
| Maximum length of a pilus | 5 μm | (3) |
| Number of pili | 1-5 | (3) |
| Pilus angle variation | Standard deviation of pili angle distribution = 0-90 (deg.) | (4) |
| Pili spring constant | $2\times10^{-5}$ N/m | |
| Pili stall force | 100 pN | (5) |
| Pili retraction velocity | 1 μm/s | (6, 7) |
| Pili elongation velocity | 1 μm/s | (6) |
| Pili attachment probability | 0.8 (*the range in (2) is 0.1-0.3, but we have used an increased value so that we can obtain a reasonable twitching velocity*) | (2) |
| Pili detachment time, τ | 4 s | (8, 9) |
| Number of cells | 1-4 | - |

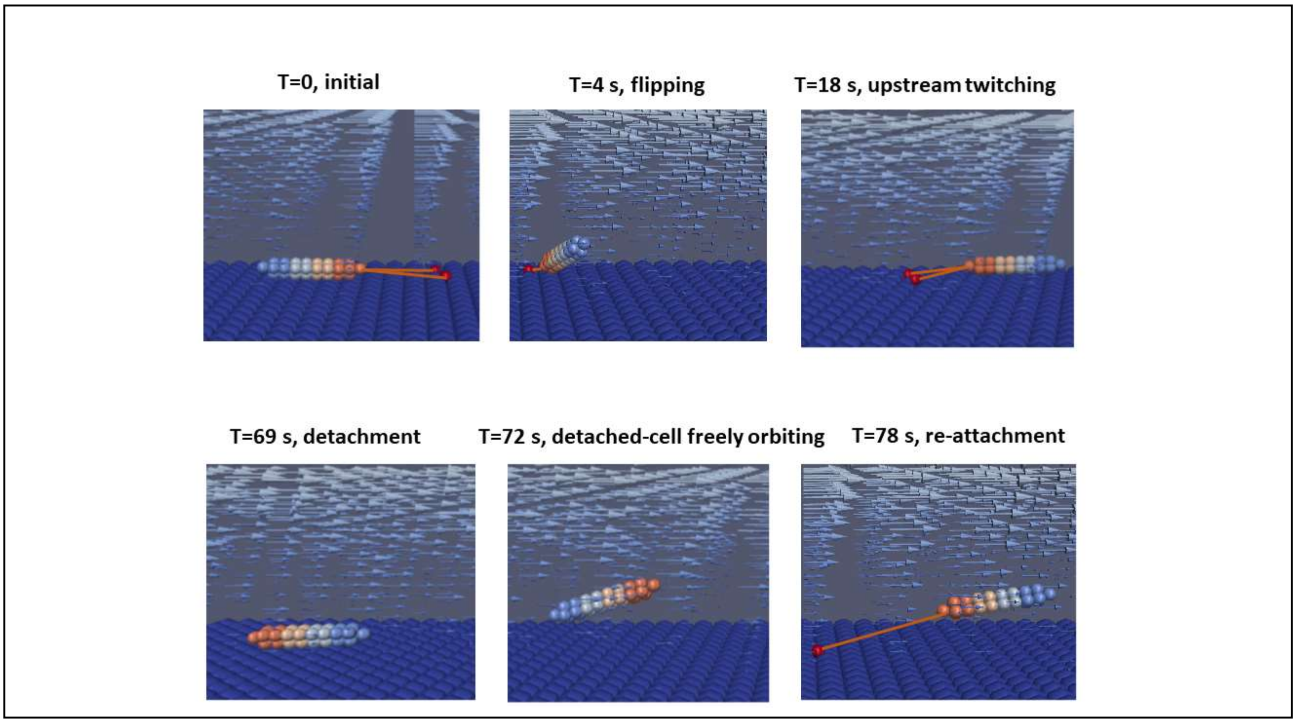


**Figure S1.** Bacterial twitching on a flat surface under shear flows.

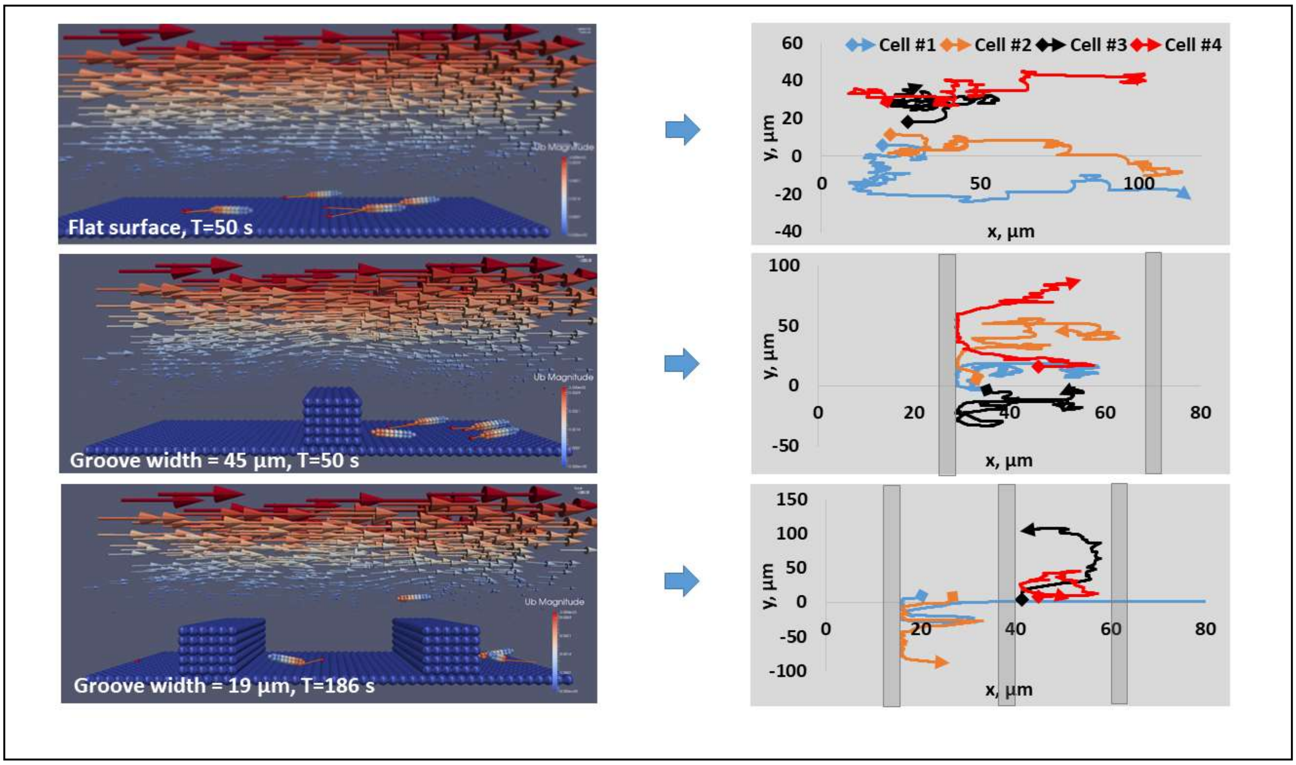


**Figure S2.** Bacterial twitching in shear flows under different groove width.

## References


1. Shen, Y., A. Siryaporn, S. Lecuyer, Z. Gitai, and H. A. Stone. 2012. Flow Directs Surface-Attached Bacteria to Twitch Upstream. Biophys J 103:146-151.
2. Zachreson, C., C. Wolff, C. B. Whitchurch, and M. Toth. 2017. Emergent pattern formation in an interstitial biofilm. Physical Review E 95.
3. Tala, L. F., A.; Kukura, P.; Persat, A. 2018. LABEL-FREE VISUALIZATION OF TYPE IV PILI DYNAMICS BY INTERFEROMETRIC SCATTERING MICROSCOPY. bioRxiv.
4. Brill-Karniely, Y., F. Jin, G. C. L. Wong, D. Frenkel, and J. Dobnikar. 2017. Emergence of complex behavior in pili-based motility in early stages of P-aeruginosa surface adaptation. Scientific Reports 7.
5. Maier, B., L. Potter, M. So, H. S. Seifert, and M. P. Sheetz. 2002. Single pilus motor forces exceed 100 pN. P Natl Acad Sci USA 99:16012-16017.
6. Skerker, J. M., and H. C. Berg. 2001. Direct observation of extension and retraction of type IV pili. P Natl Acad Sci USA 98:6901-6904.
7. Clausen, M., M. Koomey, and B. Maier. 2009. Dynamics of Type IV Pili Is Controlled by Switching Between Multiple States. Biophys J 96:1169-1177.
8. Marathe, R., C. Meel, N. C. Schmidt, L. Dewenter, R. Kurre, L. Greune, M. A. Schmidt, M. J. I. Mueller, R. Lipowsky, B. Maier, and S. Klumpp. 2014. Bacterial twitching motility is coordinated by a two-dimensional tug-of-war with directional memory. Nat Commun 5.
9. Ponisch, W., C. A. Weber, G. Juckeland, N. Biais, and V. Zaburdaev. 2017. Multiscale modeling of bacterial colonies: how pili mediate the dynamics of single cells and cellular aggregates. New J Phys 19.
